# Supplementary material for: Deep learning for enhanced risk management: a novel approach to analyzing financial reports
Source: PeerJ Comput Sci. 2025 Jan 27;11:e2661. doi: 10.7717/peerj-cs.2661 (PMC11784821; doi:10.7717/peerj-cs.2661)
Supplement: Supplemental Information 4 [file peerj-cs-11-2661-s004.docx]

To ensure reproducibility, the entire modeling process, from data preprocessing to model training and evaluation, involves the following steps:

1. **Data Splitting**: Dividing the dataset into training, validation, and test sets to ensure that the model is evaluated on unseen data.
2. **Parameter Optimization**: Using techniques such as gradient descent to optimize model parameters, ensuring that the model learns effectively from the training data.
3. **Validation**: Evaluating the model on the validation set to tune hyperparameters and prevent overfitting, which helps in improving the model's generalization to new data.

Moreover, the entire model was implemented in Python using well-established libraries such as pandas, scikit-learn, and openpyxl. The code is documented and structured to allow easy replication of the results. Additionally, the dataset and code are shared in the supplementary materials.
